# Supplementary material for: LTβR overexpression promotes plasma cell accumulation
Source: PLoS One. 2022 Aug 4;17(8):e0270907. doi: 10.1371/journal.pone.0270907 (PMC9352096; doi:10.1371/journal.pone.0270907)
Supplement: Attachment — Submitted filename: Kotov etal Response to Reviewers.docx [file pone.0270907.s002.docx]

Jessica Kotov LtbR PlosOne ms Reviewer Comments 042522

**Specific response to reviewer comments**

We thank the reviewers for their overall positive assessment of our study and for their helpful remarks.

5. Review Comments to the Author

Please use the space provided to explain your answers to the questions above. You may also include additional comments for the author, including concerns about dual publication, research ethics, or publication ethics. (Please upload your review as an attachment if it exceeds 20,000 characters)

Reviewer #1: The authors uncovered LTβR overexpression to potentially promote plasma cell accumulation.
Point to be considered:
1. While performing FACS analyses, did the authors employ unstained/isotype controls for compensation?

- For the LTbR FACS analysis we used what we hope the reviewer will agree is the most definitive negative control, cells from LTbR KO mice. For other staining panels, the specificity of the staining has been established in multiple past uses of these commercial reagents by us and others.

2. A graphical abstract/figure or panel summarizing the experimental approach would be beneficial for the reader.

- We thank the reviewer for the suggestion. We have now added summary panels to Figures 2, 4 and 5 to help explain the experimental approach, and we provide a summary model in Figure 7.

3.When discussing the methodology applied for number selection it is not clear for my understanding, how the author identify or discuss methodologically the experimental in vivo setting. Indeed, in the in vivo experiments, sample size should be calculated in a rigorous way, i.e. by using G*Power software (power of for example 80% and 0.05 statistical level, etc.). For example, assuming an effect-size of for example, 0.4 with statistical significance of α <;0.05 and a power of 80% a given number of 9 mice for each group for a total of 18 mice would be extimated. This number should then be increased to 20 considering an expected drop-out rate of 10% for the treatment. Can the authors comment on this?

- Although the reviewer indicated in response to question 2 that the statistical analysis had been performed appropriately and rigorously, we appreciate their request for more information about our decision-making for the number of mice to use when designing the experiments. We have consulted a colleague at UCSF who has a Bachelor’s degree in statistics, and he pointed out that a request for power analysis is relevant for a project proposal, but not relevant for critiquing presented data. He also pointed out that our mouse numbers are not a concern for most of the data presented because, especially when we are claiming significant differences, the effect sizes are quite large and reproducible between mice and independent experiments. While the sample size in Figure 4 experiments was low, the most important significant differences for our biological conclusions (Fig. 4C and E) have very large effect sizes and low spread.

4. Cell adhesion molecules, such as ICAM1 and JAMs, are enhanced by tumor cells in a NF-κB-dependent manner in myeloma and its microenvironment: can the authors expand this issue in the introduction/discussion section in order to boost the general interest for a broad readership from the oncology field? Indeed, As is now well known, tumors grow and evolve through a constant crosstalk with the surrounding microenvironment, and emerging evidence indicates that angiogenesis and immunosuppression frequently occur simultaneously in response to this crosstalk. Accordingly, strategies combining anti-angiogenic therapy and immunotherapy seem to have the potential to tip the balance of the tumor microenvironment and improve treatment response (refer to PMID: 32354870 and PMID: 32064051)

- We have modified the text to note that NF-kB signaling can also induce adhesion molecules and this may augment interactions in the BM microenvironment, and we cite the suggested references.

Reviewer #2: The authors provide intriguing insights regarding a partially neglected topic in MM, regarding the lymphotoxin b receptor (Ltbr) locus. LTβR has well defined roles in supporting lymphoid tissue development and function through actions in stromal and myeloid cells.
Point to be addressed:

1. Are the presented data following a normal (Gaussian) distribution? If this is the case, this should be clearly stated, and parametric tests can be performed. If this is not the case, non parametric tests should be performed instead

- Although the reviewer indicated in response to question 2 that the statistical analysis had been performed appropriately and rigorously, we appreciate their additional concern. The reviewer’s comment led us to consult with a UCSF colleague who has a Bachelor’s degree in statistics. He concluded that unpaired t-test, like we performed, are appropriate for the qPCR data and %+ or count flow cytometry summary data. These data will be approximately normally distributed. While it is not as clear that the ratio summaries of flow cytometry data (Fig 2B, 2D, 5C, and 5G) will be normally distributed, in Fig 2B, 2D, and 5C we are claiming no significant differences. Given the sample sizes and non-significance by t-test, these data will also not be significantly different by rank-sum test. The effect size in Fig. 5G is large, so rank-sum tests on these data will be significant and reflect the same conclusions that we arrived at using t-tests.

2. I would suggest to better highlight the potential limitations of this study (i.e. how would the authors translate their findings to the clinical side? The underlying message here is that more precision and pinpoint approaches needed to be tested in well designed clinical trials – a challenge, but I would be interested in their perspective of how this might be done).

- We now cite a paper that came out in *Nature* while our study was under review (new ref 23). The main message of that paper was that Ltbr over-expression in T cells could be used as an approach to drive improved effector T cell function and reduced exhaustion. We suggest that a similar engineering approach could be used to augment plasma cell generation and secreted protein production.

- We also comment that our data point to the need to consider the therapeutic activity of Mcl1 and Bcl2 inhibitors in the context of LTbR OE MM.

3. To my knowledge, the role of endothelial lymphotoxin beta receptor (LTβR) has been described to egress lymphoid organ (in this regard endothelial cells can be important as well as MM angiogenesis) and ruled out the role of LTβR from epithelial cells or dendritic cells. In this regard, BST-1, like CD38, behaves both as an ectoenzyme and signaling receptor and has been reported to regulate the trafficking of neutrophil and monocytes. Did the authors check for this immune-microenvironmental characteristic or could they envision next step aiming to compensate for this in the next future?

- We have not examined how our findings relate to CD38 ectoenzyme biology and are unsure how to connect the findings in the current study to the CD38 studies in a compelling way. We agree that this is a valuable area for future research. The possible influence of LTbR expression on other cell types, such as stromal cells, in MM is an interesting point and we now comment on this in the discussion (and cite new ref 27).

4. In the frame of point 3 thinking, CD38/LTβR could be relevant, given the importance of CD38 targeting in MM: CD31/CD38 axis activates genetic programs relevant for proliferative responses. It also indicates a contribution of this pathway to the processes mediating migration and homing. These results further support the notion that the CD31/CD38 axis is part of a network of accessory signals that modify the microenvironment, favoring localization of MM cells to growth-permissive sites (CD31 is also expressed by endothelial cells). Collectively, the authors finding might hold a theragnostic potential for CD38 targeting and overcoming drug resistance, potentially, these findings may point towards a potential Achilles’ heel of multiple myeloma that might be exploited therapeutically in the future (please refer to PMID: 31936617 and expand introduction/discussion section).

- This is an interesting point. We now comment further on the importance of BM microenvironment interactions, and we cite the indicated study (new ref 6).
